# Monocyclic Phenolic Acids; Hydroxy- and Polyhydroxybenzoic Acids: Occurrence and Recent Bioactivity Studies

**DOI:** 10.3390/molecules15117985

**Published:** 2010-11-08

**Authors:** Shahriar Khadem, Robin J. Marles

**Affiliations:** Natural Health Products Directorate, Health Products and Food Branch, Health Canada, 2936 Baseline Road, Ottawa, Ontario K1A 0K9, Canada

**Keywords:** polyphenols, phenolic acids, hydroxybenzoic acids, natural occurrence, bioactivities

## Abstract

Among the wide diversity of naturally occurring phenolic acids, at least 30 hydroxy- and polyhydroxybenzoic acids have been reported in the last 10 years to have biological activities. The chemical structures, natural occurrence throughout the plant, algal, bacterial, fungal and animal kingdoms, and recently described bioactivities of these phenolic and polyphenolic acids are reviewed to illustrate their wide distribution, biological and ecological importance, and potential as new leads for the development of pharmaceutical and agricultural products to improve human health and nutrition.

## 1. Introduction

Phenolic compounds exist in most plant tissues as secondary metabolites, *i.e*. they are not essential for growth, development or reproduction but may play roles as antioxidants and in interactions between the plant and its biological environment. Phenolics are also important components of the human diet due to their potential antioxidant activity [1], their capacity to diminish oxidative stress-induced tissue damage resulted from chronic diseases [2], and their potentially important properties such as anticancer activities [3,4,5]. 

**Figure 1:**
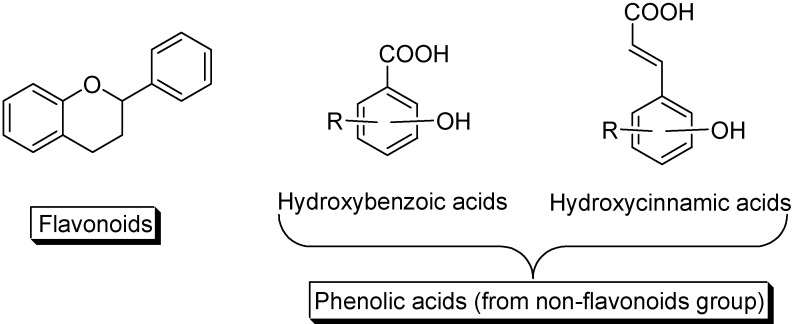
Flavonoids and phenolic acids.

The structure of phenolics consists of an aromatic ring carrying one (phenol) or more hydroxyl (polyphenol) moieties. Several classes can be distinguished according to the number of phenol rings and to the structural elements that join these rings [6]. Two main groups of polyphenols, termed flavonoids and non-flavonoid polyphenols, have been adopted in the literature [7]. The flavonoid group, including flavanones, flavones, dihydroflavonols, flavonols, flavan-3-ols, isoflavones, anthocyanidins, proanthocyanidins and chalcones, comprises those compounds with a C6-C3-C6 structure (Figure 1). 

The non-flavonoid polyphenols can be classified based on their carbon skeleton into the following subgroups: simple phenols, benzoic acids, hydrolysable tannins, acetophenones, phenylacetic acids, cinnamic acids, lignans, coumarins, benzophenones, xanthones, stilbenes, and secoiridoids. 

Phenolic acids have a carboxyl group attached or linked to benzene ring [8]. Two classes of phenolic acids can be distinguished depending on their structure: benzoic acid derivatives (*i.e.* hydroxybenzoic acids, C6-C1) and cinnamic acid derivatives (*i.e.* hydroxycinnamic acids, C6-C3) [9] (Figure 1). 

This review will cover the natural occurrence and recently described biological activities of monocyclic hydroxy- and polyhydroxybenzoic acids. Research published prior to the last ten years will not be included as considerable efforts have been made already to cover those findings [e.g., 10-12]. Many hydroxybenzoic acids have not been discussed here due to their lack of known bioactivities.

## 2. Results and Discussion

**Figure 2:**
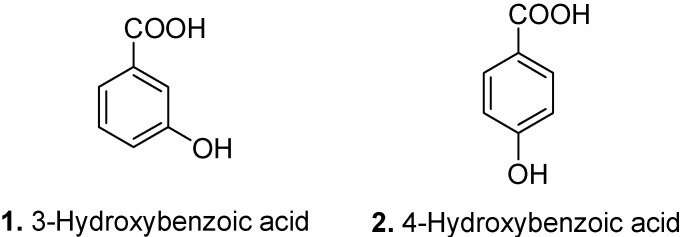
3-Hydroxybenzoic acid (**1**) and 4-Hydroxybenzoic acid (**2**).

3-Hydroxybenzoic acid (**1**, Figure 2) is found in common plants such as grapefruit (*Citrus paradisi*), olive oil (*Olea europaea*) [13], and medlar fruit (*Mespilus germanica*) [14]. It has glucosylating activity [15]. *p*-Hydroxybenzoic acid (4-hydroxybenzoic acid, **2**, Figure 2) has been isolated from many sources including carrots (*Daucus carota*) [16], oil palm (*Elaeis guineensis*) [17], grapes (*Vitis vinifera*), and numerous other species including east African satinwood (*Fagara macrophylla*), yellow-leaf tree (*Xanthophyllum rubescens*), peroba (*Paratecoma peroba*), taheebo (*Tabebuia impetiginosa*), red sandalwood (*Pterocarpus santalinus*), southern catalpa (*Catalpa bignonioides*), Chinese chastetree (*Vitex negundo*) [18], betel palm (*Areca catechu*), Cuban royal palm (*Roystonea regia*) [19], and medlar (*Mespilus germanica*) [14]. It shows antifungal, antimutagenic, antisickling, estrogenic [20], and antimicrobial [17] activities. *p*-Hydroxybenzoic acid has a growth stimulation effect on the freshwater green alga *Pseudokirchneriella subcapitata* [21].

**Figure 3:**
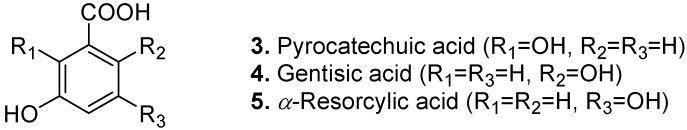
Pyrocatechuic acid (**3**), Gentisic acid (**4**), and *α*-Resorcylic acid (**5**).

Pyrocatechuic acid (2,3-dihydroxybenzoic acid, **3**, Figure 3) occurs in rhododendrons (*Rhododendron* spp.) and other members of the heather family such as winter heath (*Erica carnea*) and teaberry (*Gaultheria procumbens*), yellow gentian (*Gentiana lutea*) and the related European centaury (*Erythraea centaurium*), and the common and Madagascar or rosy periwinkles (*Vinca minor* and *Catharanthus roseus*) [22]. It is also produced by algal, bacterial and fungal microorganisms such as marine-derived actinomycetes [23], the green alga *Spongiochloris spongiosa*, the cyanobacterium *Anabaena doliolum* [24], the other bacteria *Streptomyces* sp., *Acinetobacter calcoaceticus*, *Brucella abortus*, and *Bacillus* sp., and the fungi *Aspergillus sojae*, *Rhizobium* sp. and *Penicillium roquefortii*. Pyrocatechuic acid is an antioxidant [25], a radical scavenger [23], and a siderophore [26]. It has some natural derivatives such as the 3-*O*-*β*-D-glucopyranoside isolated from totally unrelated plants such as the gentian relative *Geniostoma antherotrichum*, the common periwinkle (*Vinca minor*), and the mustard relative *Boreava orientalis*; and 2-hydroxy-3-methoxybenzoic acid from a crocus (*Colchicum decaisnei*) and a birch (*Betula pendula*).

Gentisic acid (2,5-dihydroxybenzoic acid, **4**, Figure 3) also has a widespread occurrence, being found in citrus fruits (*Citrus* spp.), grapes (*Vitis vinifera*), Jerusalem artichoke (*Helianthus tuberosus*), sesame (*Sesamum indicum*), gentians (*Gentiana* spp.), red sandalwood (*Pterocarpus santalinus*), rose gum (*Eucalyptus grandis*), saxifrage (*Saxifraga* spp.), and olive (*Olea europaea*) [13]. In addition to being an analgesic, anti-inflammatory, antirheumatic, antiarthritic, and cytostatic agent, gentisic acid inhibits low-density lipoprotein oxidation in human plasma [27]. It is believed that gentisic acid has an effective role in the anticarcinogenetic activity of China-rose hibiscus (*Hibiscus rosa-sinensis*) extract [28]. A recent study has shown that gentisic acid is a Fibroblast Growth Factor (FGF) inhibitor [29]. 

Many derivatives of gentisic acid are found naturally, such as 5-*O*-(1-carboxyethenyl) in aster (*Aster indicus*), 5-methylether in cowslip (*Primula veris*), 2-*O*-[*β*-D-glucopyranosyl-(1→3)-3-hydroxybenzoyl] in marsh felwort (*Lomatogonium rotatum*), 5-*O*-[4-hydroxy-3,5-dimethoxybenzoyl-(→5)-*β*-D-apiofuranosyl-(1→2)-*β*-D-glucopyranoside] (albizinin) in Indian albizia (*Albizia lebbek*), 5-*O*-[*β*-D-apiofuranosyl-(1→2)-*β*-D-glucopyranoside] in sensitive-plant (*Mimosa pudica*), 5-*O*-[*β*-D-apiofuranosyl-(1→2)-*β*-D-xylopyranoside] in the legume *Spatholobus suberectus*, 5-(6-galloylglucoside) in sawtooth oak (*Quercus acutissima*), 5-*O*-[4-hydroxy-3-methoxy-benzoyl-(→6)-*β*-D-glucopyranoside] in squirrel’s-foot fern (*Davallia mariesii*), 5-*O*-*β*-D-glucopyranoside in cassia (*Cassia absus*), Chinese goldthread (*Coptis chinensis*), and sensitive-plant (*Mimosa pudica*), 5-xyloside in Indian coral-tree (*Erythrina indica*), 2-*O*-*β*-D-glucopyranoside (orbicularin) in cotoneaster (*Cotoneaster orbicularis*), 5-*O*-*β*-xylopyranosyl, 5-*O*-{[5"-*O*-*E*-(4"'-*O*-threo-guaiacylglycerol)-feruloyl]-*β*-apiofuranosyl-(1→2)-*β*-xylopyranosyl}, 5-*O*-[(5"-*O*-vanilloyl)-*β*-apiofuranosyl-(1→2)-*β*-xylopyranosyl] and 1-*O*-[*E*-(4"'-*O*-threo-guaiacylglycerol)-feruloyl]-3-*O*-*β*-galacturonopyranosyl glycerol in barrel medic (*Medicago truncatula*) [30].

*α*-Resorcylic acid (3,5-dihydroxybenzoic acid, **5**, Figure 3) is a constituent of peanuts (*Arachis hypogaea*), chickpeas (*Cicer arietinum*), red sandalwood (*Pterocarpus santalinus*), and hill raspberry (*Rubus niveus*). It has nematicidal activity [31].

**Figure 4:**
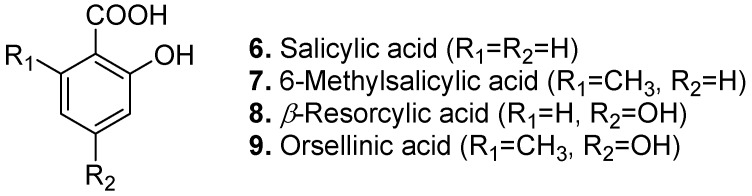
Salicylic acid (**6**), 6-Methylsalicylic acid (**7**), *β*-Resorcylic acid (**8**), and Orsellinic acid (**9**).

Salicylic acid (2-hydroxybenzoic acid, **6**, Figure 4) occurs in such diverse plants as willow bark (*Salix spp*.), poplar (*Populus pseudo-simonii*), Voodoo lily (*Sauromatum guttatum*), gumweed (*Grindelia* spp.), and medlar (*Mespilus germanica*) [14]. It is also produced by the bacterium *Pseudomonas cepacia*. Salicylic acid has keratolytic, anti-inflammatory, antipyretic, analgesic, antiseptic, and antifungal properties for several skin conditions such as dandruff and seborrhoeic dermatitis, ichthyosis, psoriasis, acne, *etc*. [32]. It functions as a hormonal mediator of plant resistance responses to environmental stress and pathogen attacks [33,34].

6-Methylsalicylic acid (2-hydroxy-6-methylbenzoic acid, **7**, Figure 4) is a polyketide derivative occurring in narrow-leaf yerba-santa (*Eriodictyon angustifolium*). It is also produced as a mold metabolite by *Phyllosticta* and *Penicillium* spp. [35]. 6-Methylsalicylic acid is a phytotoxin. It has antibacterial and antifeeding [36] activities.

*β*-Resorcylic acid (2,4-dihydroxybenzoic acid, **8**, Figure 4) is found in red sandalwood (*Pterocarpus santalinus*) and the related coralwood (*Adenanthera pavonina*). *β*-Resorcylic acid has thyroid peroxidase inhibitory effect [37]. Its methyl ether derivatives are also found naturally. For example, 2-methyl ether (pluchoic acid) is a constituent of the fleabane *Pluchea lanceolata* and 4-methyl ether is found in leaves and stems of the unrelated legume, *Anthyllis sericea*. 

Orsellinic acid (2,4-dihydroxy-6-methylbenzoic acid, **9**, Figure 4) presents in some lichens such as *Roccella*, *Lecanora*, and *Lobaria yunnanensis*. It has also been isolated from cultures of the fungi *Penicillium* spp., *Hypoxylon* spp., and *Chaetomium cochliodes*. Orsellinic acid has antimicrobial activity. Some derivatives of orsellinic acid are found naturally, for example, the 2-*O*-*β*-D-glucopyranoside in cloves (*Syzygium aromaticum*), the 2-methyl ether (isoeverninic acid) in the lichen *Lecanora gangaleoides*, and the 4-methyl ether (everninic acid) in the honey mushroom *Armillaria mellea*.

**Figure 5:**
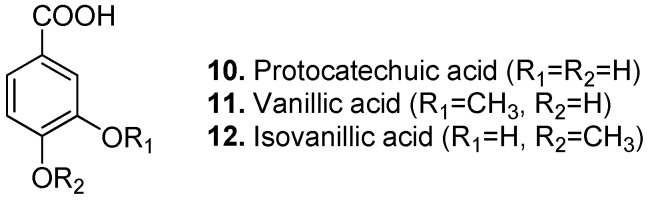
Protocatechuic acid (**10**), Vanillic acid (**11**), and Isovanillic acid (**12**).

Protocatechuic acid (3,4-dihydroxybenzoic acid, **10**, Figure 5) is found in Spanish heath (*Erica australis*), dog rose (*Rosa canina*), Korean spruce (*Picea koraiensis*), gum-tree (*Eucalyptus grandis*), the Traditional Chinese Medicine (TCM) herb shensi (*Picrorhiza kurrooa*), ferns, buckwheat (*Fagopyrum* spp.), alder (*Alnus* spp.), onion and garlic and relatives (*Allium* spp.), Japanese pepper (*Zanthoxylum piperitum*)[38], another TCM herb danshen (*Salvia miltiorrhiza*) [39], sharp-leaf galangal (*Alpinia oxyphylla*) [40], sea buckthorn (*Hippophae rhamnoides*) [41], Japanese honeysuckle (*Lonicera japonica*) [42], mulberry (*Morus alba*) [43], and medlar (*Mespilus germanica*) [14]. It has been found to have several bioactivities such as antifungal, antihepatotoxic, anti-inflammatory, antioxidant [25,44], free radical scavenger, cytotoxic [42], chemopreventive, apoptotic [45,46,47], platelet aggregation inhibitor, neuroprotective [40], and LDL oxidation inhibitor [38]. Protocatechuic acid is the major metabolite of anthocyanins [48,49]. Many protocatechuic acid glucosides are also found naturally. For example the 3-*O*-*β*-glucopyranoside is reported in lobelia (*Lobelia sessilifolia*), the 4-*O*-*β*-glucopyranoside in turnip fern (*Angiopteris lygodiifolia*) and in the oriental and American cockroaches (*Blatta orientalis* and *Periplaneta americana*) perhaps coming from their diet rather than endogenously produced, dracunculifoside B in the groundsel relative *Baccharis dracunculifolia*, and the 4-*O*-(4-*O*-methyl-*β*-D-glucopyranoside) in Japanese climbing fern (*Lygodium japonicum*).

Vanillic acid (4-hydroxy-3-methoxybenzoic acid, **11**, Figure 5) occurs in many plants such as prickly ash (*Fagara* spp.), Japanese alder (*Alnus japonica*), spiny oleaster (*Elaeagnus pungens*), Spanish heath (*Erica australis*), upland cotton (*Gossypium mexicanum*), Chinaberry (*Melia azedarach*), oriental ginseng (*Panax ginseng*), Korean peroba (*Paratecoma koraiensis*), red sandalwood (*Pterocarpus santalinus*), dog rose (*Rosa canina*), shensi (*Picrorhiza kurrooa*), luo shi (*Trachelospermum asiaticum*), ishpingo (*Amburana cearensis*), and Shiitake mushroom (*Lentinula edodes*). Besides antisickling and anthelmintic activities, vanillic acid could suppress hepatic fibrosis in chronic liver injury [50,51]. It is also found to be an inhibitor of snake venom 5'-nucleotidase [52].

Isovanillic acid (3-hydroxy-4-methoxybenzoic acid, **12**, Figure 5) is a methyl ether derivative of protocatechuic acid. It is found in hortensia (*Hydrangea macrophylla*), Chinese endospermum tree (*Endospermum chinense*) [53], the orange relative *Citrus changshan-huyou* [54], Chinese banyan (*Ficus microcarpa*) [55], the chamomile relative *Anthemis melanolepis* [56], poonspar (*Calophyllum polyanthum*) [57], sanchi ginseng (*Panax notoginseng*) [58], Formosa koa (*Acacia confusa*) [59,60], the breadfruit relative *Treculia obovoidea* [61], and saffron (*Crocus sativus*) [62]. Isovanillic acid has antibacterial [56,61] and antioxidant [59,60] activities.

**Figure 6:**
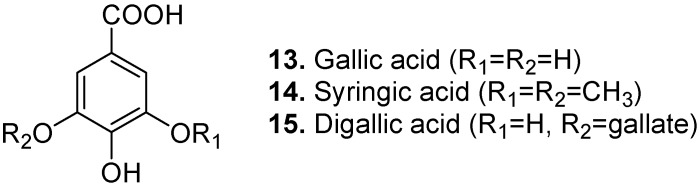
Gallic acid (**13**), Syringic acid (**14**), and Digallic acid (**15**).

Gallic acid (3,4,5-trihydroxybenzoic acid, **13**, Figure 6) is a widespread phytochemical that occurs in tallow-tree (Allanblackia floribunda), the mangosteen relative Garcinia densivenia, bridelia (Bridelia micrantha), sappanwood (Caesalpinia sappan), elephant-apple (Dillenia indica), cinnabar ebony (Diospyros cinnabarina), peroba (Paratecoma peroba), guava (Psidium guajava), water-berry (Syzygium cordatum), staghorn sumac (Rhus typhina), tamarisk (Tamarix nilotica), grape (Vitis vinifera), witch-hazel (*Hamamelis virginiana*) [63], and red toon (*Toona sinensis*) [64]. It has uses as an astringent and styptic. Besides having antineoplastic and bacteriostatic activities, gallic acid possesses antimelanogenic and antioxidant properties [65]. A phenolic fraction from evening primrose (Oenothera biennis) containing gallic acid showed anti-tumour activity [66]. Gallic acid has shown anticancer properties in prostate carcinoma cells [64,67,68]. It is believed that gallic acid is partially responsible for the antiangiogenic activities of sweet leaf tea (*Rubus suavissimus*) extract [69]. Gallic acid is a potent inhibitor of brush border sucrase and other disaccharidases in the mammalian intestine [70]. It showed promising results as an anti-HSV-2 (Herpes simplex virus) agent [71]. Gallic acid has been proposed to be a candidate for treatment of brain tumours as it suppresses cell viability, proliferation, invasion, and angiogenesis in human glioma cells [72], although the cytotoxic effects of tannins are generally not specific to tumour cells. Gallic acid induced HeLa cervical cancer cells death via apoptosis and/or necrosis [73]. Many gallic acid derivatives (as phenolic acids) are naturally occurring. This includes 3-*O*-*β*-D-glucopyranoside (3-glucogallic acid) from rhubarb (*Rheum* spp.), 3-*O*-(6-galloylglucoside) from rhubarb and great burnet (*Sanguisorba officinalis*), 3-*O*-[*β*-D-apiofuranosyl-(1→6)-*β*-D-glucopyranoside] (or mudanoside B) from tree peony (*Paeonia suffruticosa*), 4-*O*-(6-galloylglucoside) from rhubarb, 3-*O*-dodecanoyl (3-lauroylgallic acid) with antioxidant and antimicrobial activities from the palm tree *Satakentia liukiuensis*, 3-methyl ether from the geranium *Geranium collinum* and the knotweed relative mu liao (*Atraphaxis frutescens*), 3-methyl-5-*O*-sulfate (as salts) from sea-heath (*Frankenia laevis*) and tamarisk (*Tamarix amplexicaulis*), 3-methyl-4-*O*-[3,4-dihydroxy-5-methoxybenzoyl-(→6)-*β*-D-glucopyranoside] (or bistortaside A) from bistort (*Polygonum bistorta*), 3-methyl-5-*O*-*β*-D-glucopyranoside from the dogbane relative *Tabernaemontana cymosa*, 3-methyl ether from the cashew relative *Poupartia axillaris* and the related smooth sumac (*Rhus glabra*), 3-ethyl ether from emblic (*Phyllanthus emblica*), and 4-ethyl ether from mimosas (*Mimosa hamata*, *Mimosa rubicaulis*), logwood (*Haematoxylum campechianum*), strawberry-tree (*Arbutus unedo*), cider tree (*Eucalyptus gunnii*), black myrobalan (*Terminalia chebula*) and the toxic legume *Elephantorrhiza elephantina*.

Syringic acid (4-hydroxy-3,5-dimethoxybenzoic acid, **14**, Figure 6) occurs in many natural sources including Chinese catalpa (*Catalpa ovata*), garden balsam (*Impatiens balsamina*), New Jersey tea (*Ceanothus americanus*), *Citrus* spp., soybean (*Glycine max*), saxifrages (Saxifragaceae), thyme (*Thymus vulgaris*), summer savory (*Satureja hortensis*), hyssop (*Hyssopus officinalis*), rosemary (*Rosmarinus officinalis*) [74], pot marigold (*Calendula officinalis*) [75], tinder fungus (*Phellinus igniarius*) [76], golden eye grass (*Curculigo orchioides*) [77], date (*Phoenix dactylifera*) [78], sea hibiscus (*Hibiscus tiliaceus*) [79], Natal mahogany (*Trichilia emetica*) [80], birch conk (*Inonotus obliquus*) [81], chickory (*Cichorium intybus*) [82], finger millet (*Eleusine coracana*) [83], woad (*Isatis tinctoria*) [84], clove (*Syzygium aromaticum*) [85], shiitake (*Lentinula edodes*) [50], the African medicinal shrub *Anisophyllea dichostyla* [86], French tamarisk (*Tamarix gallica*) [87], the Brazilian medicinal tree *Caraipa densifolia* [88], propolis (resinous materials gathered by bees from tree buds, sap flows and various other botanical sources, obtained in this case from Turkey) [89], rhododendrons (*Rhododendron* spp.) [90], medlar (*Mespilus germanica*) [14], and several other cereal grains such as barley, maize, millet, oat, rice, rye, sorghum, and wheat [91]. Besides being an antioxidant, syringic acid has antibacterial [84] and hepatoprotective [50,51] activities.

Digallic acid ([3,4-dihydroxy-5-[(3,4,5-trihydroxybenzoyl)oxy]benzoic acid], **15**, Figure 6) is isolated from sweet acacia (*Acacia farnesiana*), gum arabic (*Acacia arabica*), dawn redwood (*Metasequoia glyptostroboides*), chinkapin (*Castanopsis* spp.), oriental white oak (*Quercus aliena*) [92], mango (*Mangifera indica*) [93], Chinese sumac (*Rhus chinensis*) [94], wild granadilla (*Adenia cissampeloides*) [95], black myrobalan (*Terminalia chebula*) [96], and mastic (*Pistacia lentiscus*) [97]. It is an HIV reverse transcriptase inhibitor. Digallic acid has cytotoxic/antiapoptotic activity [3]. It also shows antigenotoxic and antioxidant activities [97].

**Figure 7:**
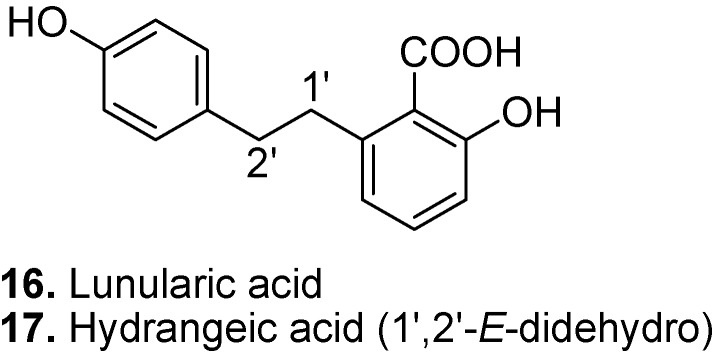
Lunularic acid (**16**) and Hydrangeic acid (**17**).

Lunularic acid ([2-hydroxy-6-[2-(4-hydroxyphenyl)ethyl]benzoic acid], **16**, Figure 7) has been isolated from hortensia (*Hydrangea macrophylla*), the liverworts *Lunularia cruciata* [98], *Riella spp*., *Marchantia polymorpha*, *Blasia pusilla,* and *Riccia spp*. [99], and celery (*Apium graveolens*) [100]. It has growth inhibitory and dormancy-inducing effects for lower plants [101]. It has also shown fungicidal, algicidal and antihyaluronidase activities [102].

Hydrangeic acid ([2-hydroxy-6-[2-(4-hydroxyphenyl)ethenyl]benzoic acid], **17**, Figure 7) is a stilbenecarboxylic acid constituent of hortensia (*Hydrangea macrophylla*) [98]. Hydrangeic acid possesses anti-diabetic activity and lowers blood glucose, triglyceride and free fatty acid levels [103].

**Figure 8:**
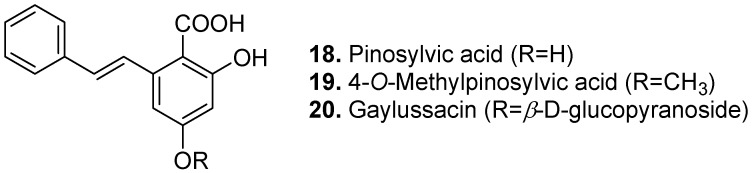
Pinosylvic acid (**18**), 4-*O*-Methylpinosylvic acid (**19**), and Gaylussacin (**20**).

Pinosylvic acid ([2,4-dihydroxy-6-styrylbenzoic acid], **18**, Figure 8) is another stilbenecarboxylic acid found in climbing skullcap (*Scutellaria scandens*). The leaves of this plant are traditionally used to treat wounds and swelling by insects [104]. 

4-*O*-Methylpinosylvic acid (2-hydroxy-4-methoxy-6-styrylbenzoic acid, **19**, Figure 8) is the methyl ether derivative of pinosylvic acid found in leaves of pigeon pea (*Cajanus cajan*) [105]. The 4-*O*-β-D-glucopyranoside derivative of pinosylvic acid, called gaylussacin (**20**, Figure 8), is found in black huckleberry (*Gaylussacia baccata*), dangleberry (*Gaylussacia frondosa*) and climbing skullcap (*Scutellaria scandens*) [106]. 

**Figure 9:**
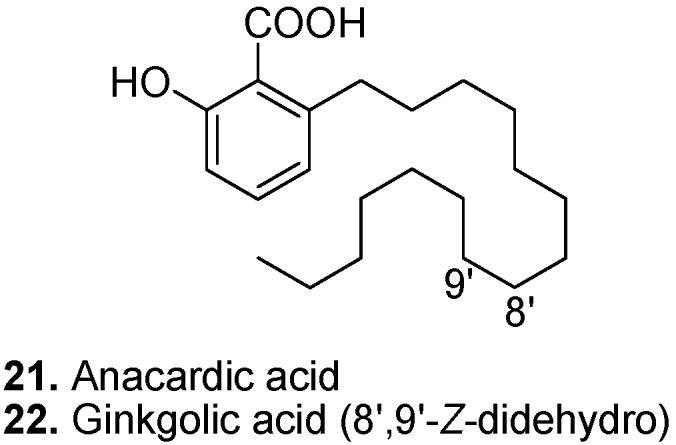
Anacardic acid (**21**) and Ginkgolic acid (**22**).

Anacardic acid (6-pentadecyl-2-hydroxybenzoic acid, **21**, Figure 9) is a phenolic lipid; one of the 6-alkylated-2-hydroxybenzoic acids. The name “anacardic acid” is also used for a mixture of different 6-alkylated-2-hydroxybenzoic acids in which the alkyl chain is either saturated or unsaturated. Anacardic acid is found in cashew (*Anacardium occidentale*) [107], ginkgo (*Ginkgo biloba*), sumac (*Rhus javanica*), zonal geranium (*Ozoroa mucronata*), pistachio (*Pistacia vera*), the Thai medicinal tree *Knema elegans*, heart-leaf philodendron (*Philodendron scandens*), California figwort (*Scrophularia californica*), and cuachalalate (*Amphipterygium adstringens*). A mixture of anacardic acids showed antibacterial action against methicillin-resistant *Staphylococcus aureus* (MRSA) [108]. Some anacardic acids have also been found to be lipoxygenase inhibitors [109]. Anacardic acids prevent generation of superoxide radicals by inhibiting xanthine oxidase [110]. Anacardic acid has bioactivity against Colorado potato beetle (*Leptinotarsa decemlineata*) larvae [111]. An anacardic acid mixture has shown anti-*Helicobacter pylori* activity [112].

Ginkgolic or ginkgoic acid ([2-hydroxy-6-(8-pentadecenyl)benzoic acid], **22**, Figure 9) is a derivative of anacardic acid isolated from ginkgo [113,114] and cashew [115]. Besides antitumor and antitubercular activities, ginkgolic acid inhibits protein SUMOylation. Small ubiquitin-related modifier proteins (SUMO) control several cellular functions, which can be related to cancer and neurodegenerative diseases [116].

**Figure 10:**
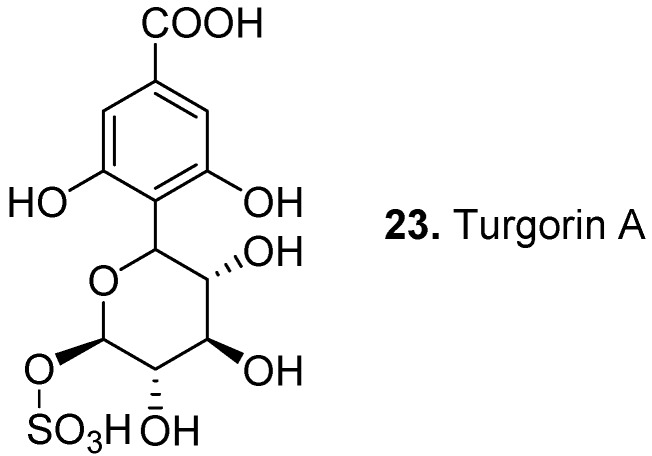
Turgorin A (**23**).

Turgorins such as turgorin A (**23**, Figure 10) are Periodic Leaf Movement Factor (PLMF) substances isolated from honey locust (*Gleditsia triacanthos*) (PLMF1, PLMF3-6), karoo-thorn (*Acacia karroo*) (PLMF1-2), sensitive-plant (*Mimosa pudica*) (PLMF1), yellow wood-sorrel (*Oxalis stricta*) (PLMF3), silk tree (*Albizia julibrissin*) (K-PLMF1), black locust (*Robinia pseudoacacia*), and hairy Indian mallow (*Abutilon grandifolium*). They are believed to be substances that control thigmonastic (touch-sensitive) and nyctinastic (diurnal light and temperature-sensitive) leaf movements [117]. Recent studies have shown that nyctinastic leaf movement is not regulated by plant hormones but rather by chemicals that differ depending on the plant species [118,119]. For example, the potassium salt of PLMF1 is the leaf-closing substance for *Mimosa pudica* [120].

**Figure 11:**
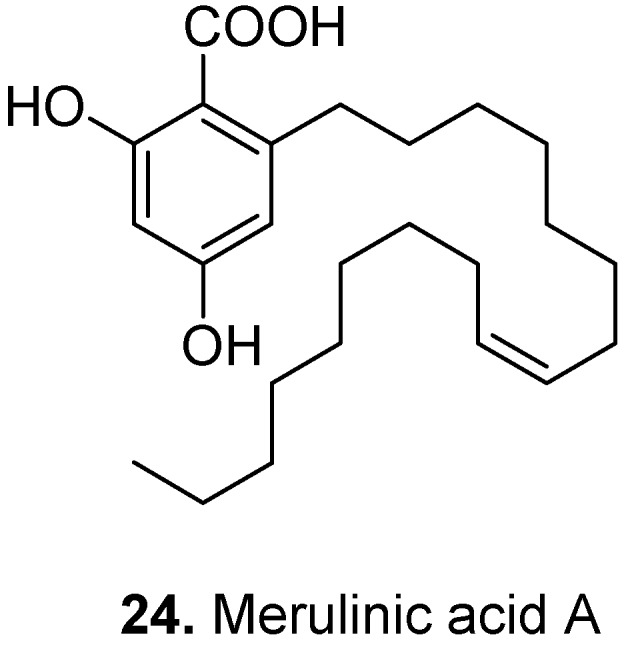
Merulinic acid A (**24**).

Merulinic acid A (**24**, Figure 11) is a phenolic lipid isolated from basidiomycetes such as *Hapalopilus mutans* [121], *Phlebia radiata*, and *Merulius tremellosus*. It has antibacterial activity, for example against *Arthrobacter citreus*, *Bacillus subtilis*, *Corynebacterium insidiosum*, *Micrococcus roseus*, and *Sarcina lutea* [122]. Merulinic acid A has pronounced promotory and/or inhibitory activities on biological membranes as an amphiphilic molecule [123].

**Figure 12:**
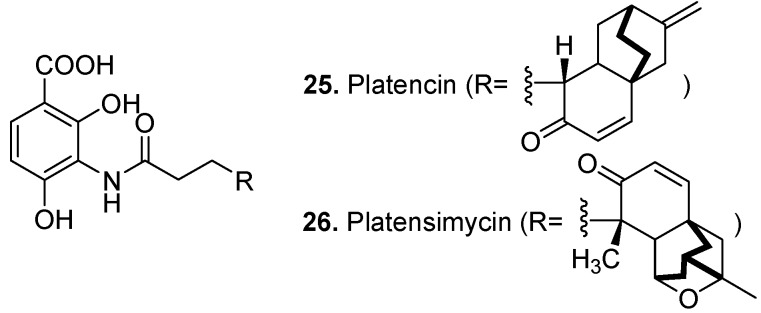
Platencin (**25**) and Platensimycin (**26**).

Platencin (**25**, Figure 12) and its analogs (platencin A_1_-A_4_) were isolated from the bacterium *Streptomyces platensis* [124,125,126]. They have been found to be dual FabF and FabH inhibitors of bacterial fatty acid biosynthesis enzymes, dubbed ‘Superbug challengers’ [127]. Superbugs are bacteria resistant to almost all antibiotics. Platencin shows broad-spectrum antibacterial activity against gram-positive pathogens such as *S. aureus*, MRSA, macrolide- and Linezolid-resistant *S. aureus*, Vancomycin intermediate *S. aureus*, Vancomycin-resistant *enterococci* and *Streptococcus pneumonia* [128]. 

Platensimycin (**26**, Figure 12) is another superbug challenger produced by *Streptomyces platensis* isolated from soil [129,130]. Platensimycin is an inhibitor of cellular lipid biosynthesis and active against gram-positive bacteria including MRSA [131,132].

**Figure 13:**
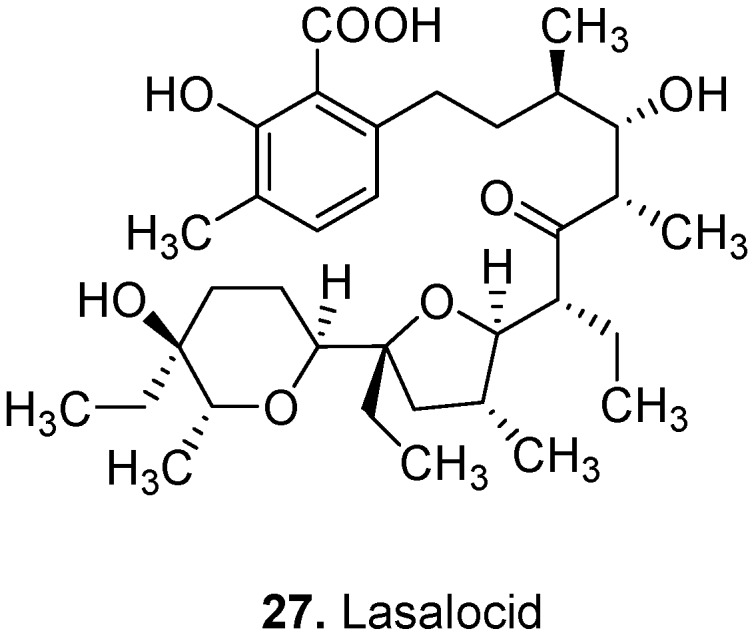
Lasalocid (**27**).

Lasalocid (Lasalocid A, **27**, Figure 13) is an ionophorous (transport-inducing) [133] antibiotic produced by *Streptomyces lasaliensis*. Its sodium salt is used as an antiprotozoal in veterinary practice for the prevention of coccidiosis [134].

**Figure 14:**
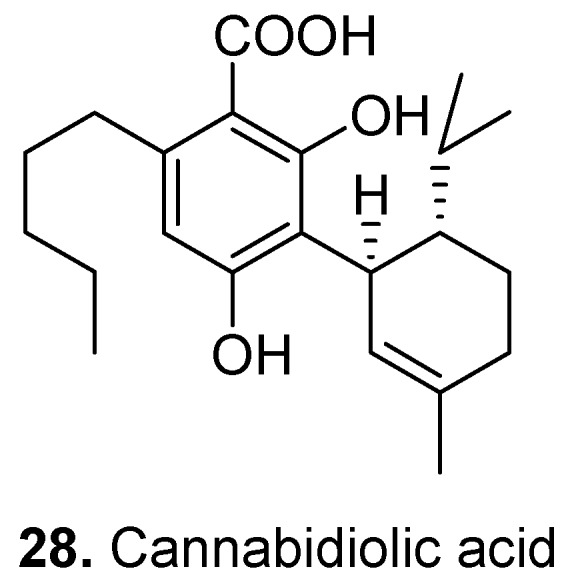
Canabidiolic acid (**28**).

Cannabidiolic acid (**28**, Figure 14) is a cannabinoid from marijuana (*Cannabis sativa*) [135,136]. It is a selective cyclooxygenase-2 inhibitor [137], TRPA1 (a member of the transient receptor potential channel family) and TRPV1 (a member of the transient receptor potential vanilloid family) agonist and TRPM8 (a member of the transient receptor potential cation channel family) antagonist [138]. Cannabidiolic acid exerts anti-proliferative actions [139].

**Figure 15:**
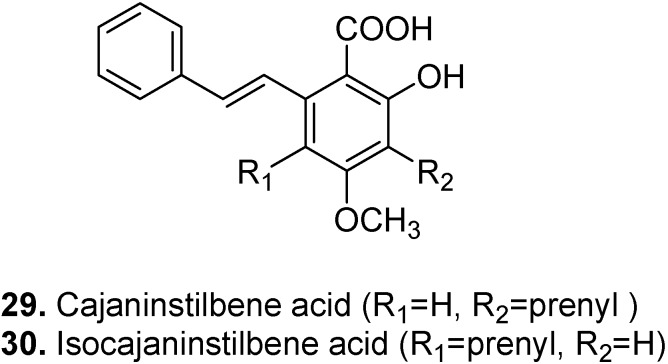
Cajaninstilbene acid (**29**) and Isocajaninstilbene acid (**30**).

Cajaninstilbene acid (3-hydroxy-4-prenyl-5-methoxystilbene-2-carboxylic acid, **29**, Figure 15) is a stilbenecarboxylic acid found in pigeon pea (*Cajanus cajan*) [140]. It has hypotriglyceridic and hypoglycaemic activities [141,142]. Besides being a good antioxidant [143,144], cajaninstilbene acid has potential use in the treatment of postmenopausal osteoporosis [145]. It also showed anti-inflammatory, impermeability (not permitting fluids to pass through) and analgesic effects [146].

Isocajaninstilbene acid (6-hydroxy-4-methoxy-3-prenyl-2-styrylbenzoic acid, **30**, Figure 15) is an isoprenylated stilbene-2-carboxylic acid also found in the leaves of pigeon pea [105,147].

## 3. Conclusions

The structural features common to the 30 compounds described in this review are the presence of benzoic and phenolic functional groups on a core monocyclic carbon skeleton. This does not imply a common biosynthetic origin. Many of these compounds arise from the shikimic acid pathway that starts with the coupling of phosphoenolpyruvate and D-erythrose-4-phosphate to give the core 6-membered ring with one carboxyl and three hydroxyl substituents. However, other molecules with similar functionality, such as the orsellinic acids, cannabidiolic acid and 6-methylsalicylic acid, are biosynthesized through the acetate pathway via polyketide intermediates. This indicates that the source organisms have a variety of routes by which these monocyclic phenolic acids can be synthesized.

By providing detailed descriptions of the source organisms for these monocyclic phenolic acids, we have endeavored to demonstrate that unlike many secondary metabolites which have a very restricted distribution in the bacterial, algal, fungal, and plant (and to a much lesser and generally secondary extent, animal) kingdoms, many of the compounds discussed here are found in a wide diversity of unrelated plant, algal, fungal, and bacterial species. Since, as secondary metabolites, their biosynthesis arises from mutations in the genes coding for enzymes involved in the biosynthesis of primary metabolites, a wide distribution in distantly related or unrelated species suggests that the mutations occurred early in phylogeny and are highly conserved and/or they occurred more recently and frequently across the taxa, and have been conserved. In either case, their frequent occurrence suggests that many of these phenolic acids confer advantages to the survival of the source organisms.

Despite their various biosynthetic origins, many of these molecules have been shown in experimental studies to have similar biological functions. For example, they have antioxidant, antimutagenic and even leaf movement regulating agents that protect the organism that produces them from the oxidative stress created by metabolism and their physical environment. They also have antiviral, antibacterial (bactericidal, bacteriostatic), algicidal, plant growth regulating, phytotoxic, antifungal, antiprotozoal, nematicidal, insecticidal, antifeedant, and mammalian estrogenic, keratolytic, platelet aggregation inhibiting, hypoglycemic, cytotoxic, and neurotoxic activities that may serve to protect the organism that biosynthesizes them from competing, pathogenic, and herbivorous organisms in their biological environment.

The diverse biological functions of these monocyclic phenolic acids suggest potential pharmacological activities. Thus, this review of the structures, occurrence and activities of phenolic acids can provide not only ecological insights but leads for the development of natural and derivative pharmaceutical and agricultural chemicals with implications for significant benefits to human health and nutrition.

The focus of this review on the last 10 years of peer-reviewed publications has shown that the study of the chemistry, occurrence, biological and pharmacological functions of the monocyclic phenolic acids continues to be a very active and dynamic field of investigation. From this it is reasonable to predict that many novel compounds and applications remain to be discovered.
